# Personalized Virtual Reality Compared With Guided Imagery for Enhancing the Impact of Progressive Muscle Relaxation Training: Pilot Randomized Controlled Trial

**DOI:** 10.2196/48649

**Published:** 2024-01-30

**Authors:** Susanna Pardini, Silvia Gabrielli, Silvia Olivetto, Francesca Fusina, Marco Dianti, Stefano Forti, Cristina Lancini, Caterina Novara

**Affiliations:** 1 Department of General Psychology University of Padova Padova Italy; 2 Digital Health Research, Centre for Digital Health and Wellbeing Fondazione Bruno Kessler Trento Italy; 3 Human Inspired Technology Research Centre (HIT) University of Padova Padova Italy; 4 Padova Neuroscience Center University of Padova Padova Italy; 5 Centre for Digital Health and Wellbeing Fondazione Bruno Kessler Trento Italy

**Keywords:** digital health, progressive muscular relaxation technique, mental well-being, virtual reality therapy, anxiety, relaxation, e-therapy, eHealth, mobile phone

## Abstract

**Background:**

Empirical evidence has shown that virtual reality (VR) scenarios can increase the effects of relaxation techniques, reducing anxiety by enabling people to experience emotional conditions in more vivid settings.

**Objective:**

This pilot randomized controlled study aims to investigate whether the progressive muscle relaxation technique (PMRT) associated with a personalized scenario in VR promotes psychological well-being and facilitates the recall of relaxing images more than the standard complementary intervention that involves the integration of PMRT and guided imagery (GI).

**Methods:**

On the basis of a longitudinal, between-subject design, 72 university students were randomly exposed to one of two experimental conditions: (1) standard complementary procedure (PMRT and GI exposure) and (2) experimental procedure (PMRT and personalized VR exposure). Individuals were assessed by a therapist before and after 7 training sessions based on measures investigating anxiety, depression, quality of life, coping strategies, sense of presence, engagement, and side effects related to VR exposure. Heart rate data were also collected.

**Results:**

Differences in changes between the 2 groups after the in vivo PMRT session conducted by the psychotherapist (T1) were statistically significant for state anxiety (*F*_1,67_=30.56; *P*<.001) and heart rate (*F*_1,67_=4.87; *P*=.01). Individuals in the VR group obtained lower scores both before (*t*_67_=−2.63; *P*=.01; Cohen *d*=0.91) and after (*t*_67_=−7.23; *P*<.001; Cohen *d*=2.45) the relaxation session when it was self-administered by participants (T2). A significant reduction in perceived state anxiety at T1 and T2 was observed for both groups (*P*<.001). After the VR experience, individuals reported feeling higher engagement in the experience than what was mentioned by participants in the GI group (*F*_1,67_=2.85; *P*=.03; η_p_^2^=0.15), and they experienced the environment as more realistic (*F*_1,67_=4.38; *P*=.003; η_p_^2^=0.21). No differences between groups regarding sense of presence were found (*F*_1,67_=1.99; *P*=.11; η_p_^2^=0.11). Individuals exposed before to the VR scenario (T1) referred to perceiving the scenario recalled in-imagination at T2 as more realistic than what those in the GI group experienced (*F*_1,67_=3.21; *P*=.02; η_p_^2^=0.12). The VR group had lower trait anxiety levels than the GI group after the relaxation session during session 7 (T2; *t*_67_=−2.43; *P*=.02).

**Conclusions:**

Personalized relaxing VR scenarios can contribute to improving relaxation and decreasing anxiety when integrated with PMRT as a complementary relaxation method.

**Trial Registration:**

ClinicalTrials.gov NCT05478941; https://classic.clinicaltrials.gov/ct2/show/NCT05478941

**International Registered Report Identifier (IRRID):**

RR2-10.2196/44183

## Introduction

### Background

In working adults and university students, the prevalence of depression, anxiety, and stress increases, predisposing them to physical diseases and general repercussions on well-being [1,2].

The situation has worsened owing to the COVID-19 outbreak, which affected individuals’ general well-being worldwide [3,4]. Different types of standardized relaxation interventions (eg, mindfulness-based stress reduction, progressive relaxation techniques by Jacobson, autogenic training by Schultz, abdominal relaxations, and visualizations) before, during, and after the COVID-19 pandemic have been shown to reduce anxiety and stress symptomatology in university students [4,5], with a more significant effect when integrated with other complementary techniques such as guided imagery (GI) [6,7]. GI is useful in creating mental imagery and refocusing attention on pleasant and relaxing imagined visual, auditory, tactile, or olfactory sensations, resulting in specific psychological and physiological responses such as relaxation and reduction of the autonomic nervous system responses [6,8,9].

The integration of the progressive muscle relaxation technique (PMRT) and GI can promote a higher sense of relaxation during training sessions, allowing for the application of the complementary techniques to cope with the stress experienced in daily activities [6,10-14].

Virtual reality (VR) is a usable, engaging, and user-friendly technology that promotes full immersion in the virtual context and facilitates the control of disturbing external stimuli [15,16].

Various studies have demonstrated that being immersed in virtual natural environments through a head-mounted display (HMD) facilitates the reduction of anxiety and stress symptoms in college students [17] as well as in association with different types of relaxation training (eg, body scan and muscle relaxation training) [18-21].

Owing to the positive impact of GI with PMRT in promoting relaxation, it may be hypothesized that exposure to a more vivid, closer-to-reality VR experience could be a helpful strategy for improving the relaxation learning promoted by the PMRT and decreasing stress and anxiety symptomatology.

GI offers an undefined number of possibilities for personalizing the imagined scenarios in a safe context. The personalization of content in VR should be helpful in recreating situations close to users’ needs to promote relaxation and can override the limits of GI. The personalization of VR environments is a crucial element to consider as it allows for relaxation and the perception of safety in the virtual context [18,22] owing to the possibility of offering a more realistic emotional experience, reflecting the users’ needs [23].

Furthermore, the first part of the PMRT, named “active PMRT,” involves a series of active sessions in which people learn how to tense and release the different muscle groups from the bottom to the top of the body to recognize the subjective state of muscle relaxation and relax the muscle areas of the body, reducing tension interfering with skeletal muscle activity.

These previous active PMRT phases are essential and are typically administered face-to-face by a psychotherapist or health operator trained to conduct the PMRT. By considering the effective results of web-based interventions in reducing stress among college students [24] and the relevant advantages that web-based therapy can offer (eg, saving costs related to attending psychotherapy sessions, allowing for partial self-management of the relaxation training), we assumed that assessing the efficacy of PMRT in alternative settings may facilitate the administration of treatment when the implementation of the standard procedure is not possible.

In light of the evidence described, we hypothesized that VR is more effective than in-imagination exposure in allowing for relaxation and decreasing state anxiety because of a more realistic sensory experience, facilitating visualization.

### Objectives

For this reason, our primary aim was to deploy the active PMRT sessions remotely via Zoom (Zoom Video Communications) and the last complementary session in the therapist’s presence, exposing people to a passive progressive relaxation session with GI or VR.

More in detail, we investigated whether PMRT associated with a personalized relaxing scenario in VR can be more effective in reducing state anxiety, tension or activation, and heart rate.

The secondary purposes of this research pilot were to (1) understand whether VR promotes a better sense of presence and engagement in the scenario compared with GI after session 6 (T1) and whether it helps recall the image and be immersed in the relaxing scenario in session 7 (T2) and (2) investigate whether exposure to the VR scenario promotes a better perception of psychological well-being, stress, and trait anxiety symptoms.

## Methods

### Overview

This pilot study was a randomized, parallel-assignment, open-label, controlled, single-center trial based on a longitudinal, between-subject design conducted at the University of Padova (Italy) in collaboration with the Center for Digital Health and Wellbeing–Fondazione Bruno Kessler (Italy). This study is part of a larger research protocol published by Pardini et al [25] (International Registered Report Identifier: RR2-10.2196/44183). The study’s recruitment phase ended in February 2023.

### Participants

Before study enrollment, during the T0 face-to-face assessment phase at the university, the participants signed a written informed consent form based on a paper-and-pencil form agreeing to participate in all the study sessions. They were informed that (1) their data would be confidential, (2) they could omit any information they did not wish to provide, and (3) they could withdraw from the study without explanation.

Study participants were recruited in Northeast Italy via social networking websites (on the web) and during university lectures (offline). Those individuals interested in participating were asked to attend a face-to-face assessment with the investigators to participate in the first evaluation phase (T0) to comply with the inclusion and exclusion criteria, provide written informed consent, and undergo a baseline evaluation.

Eligible participants were adults from the general population (aged ≥18 years) and native Italian speakers, owned a PC, and were able to use a PC and a smartphone.

Participants were excluded from the study if they had been diagnosed with a severe mental disorder or medical conditions (eg, neuromuscular disorders) or were undergoing current psychotherapeutic treatment.

Eligible participants were randomly allocated to one of the experimental conditions based on a simple blinded randomization via an Excel (Microsoft Corp) file using the “RAND” function. In total, 2 experimenters trained in cognitive and behavioral therapy conducted the relaxation sessions in a balanced way, each administering the sessions to 50% of the sample of each group to control for possible biases related to the therapist’s personality and competencies. During the allocation to one of the experimental conditions, participants were unaware of whether they had to undergo the trial in the VR or GI condition. Participants were then informed about the type of procedure. The random allocation sequence and participant enrollment were conducted by 2 experimenters trained in cognitive and behavioral therapy.

### Intervention

#### Hardware and Software Equipment

A commercially available VR headset (Meta Quest 2; Reality Labs) with an Alienware m15 Ryzen Edition R5” workstation and a link cable were used. The virtual scenarios were developed using the Unity framework (Unity Technologies) and the C# programming language (source of assets: Freesound [26], Unity, Poly Haven, and HDRIs). The code was versioned via GitLab. More detailed information about the virtual environment design is provided in the study by Pardini et al [18].

#### Procedure

**Figure 1:**
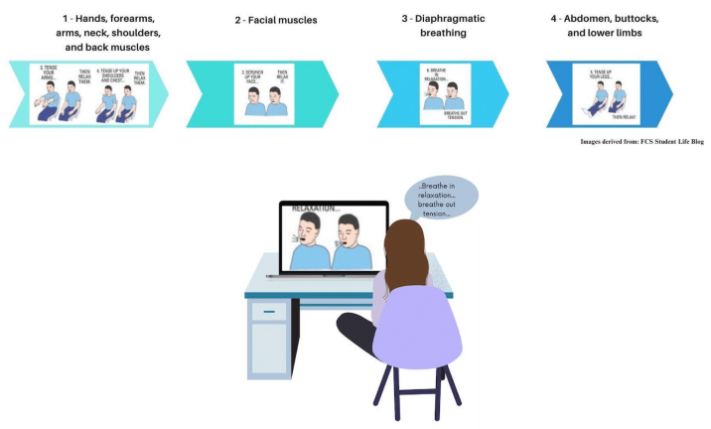
Illustrative examples of the relaxation exercises during the 4 web-based active progressive muscle relaxation technique sessions.

For each group, the intervention implied previous learning of the adapted abbreviated PMRT [27] in 4 web-based active PMRT sessions. Each session required approximately 25 minutes (Figure 1). To check whether participants completed the active PMRT sessions, the therapists conducting the experimental procedure could check whether participants completed the assessment phases and the active PMRT sessions accessing Moodle (Moodle HQ). Therapists sent a reminder to participants a day before each session in the form of a direct message from the Moodle platform that was received as a personal email on the webmail university platform.

The first session focused on tensing and releasing the hands, forearms, arms, neck, shoulders, and back muscles; the second session focused on the facial muscles; the third session focused on teaching diaphragmatic breathing; and the fourth session focused on the abdomen, buttocks, and lower limbs (Figure 1).

The web-based sessions took place on the Zoom platform and were conducted by a cognitive behavioral therapist. Each Zoom session consisted of (1) filling out the State-Trait Anxiety Inventory–Form Y1 (STAI-Y1; state anxiety form), (2) sharing general standardized instructions for relaxation and the PMRT background with the participant, and (3) active PMRT session focused on a particular body section.

The fifth VR session was conducted at the VR laboratory at the University of Padova. All participants were asked to wear the Xiaomi 2 smartwatch to detect their heart rate frequency before, during, and after the relaxation session. A total of 5 measurements (1 per minute) were taken before the relaxation experience, 12 were taken during the entire exposure, and 5 were taken after the experience in the virtual context. Both the PMRT with GI and the PMRT with VR sessions were approximately 12 minutes long. This duration was also established based on previous studies’ outcomes and to avoid potential cybersickness symptoms [28,29].

**Figure 2:**
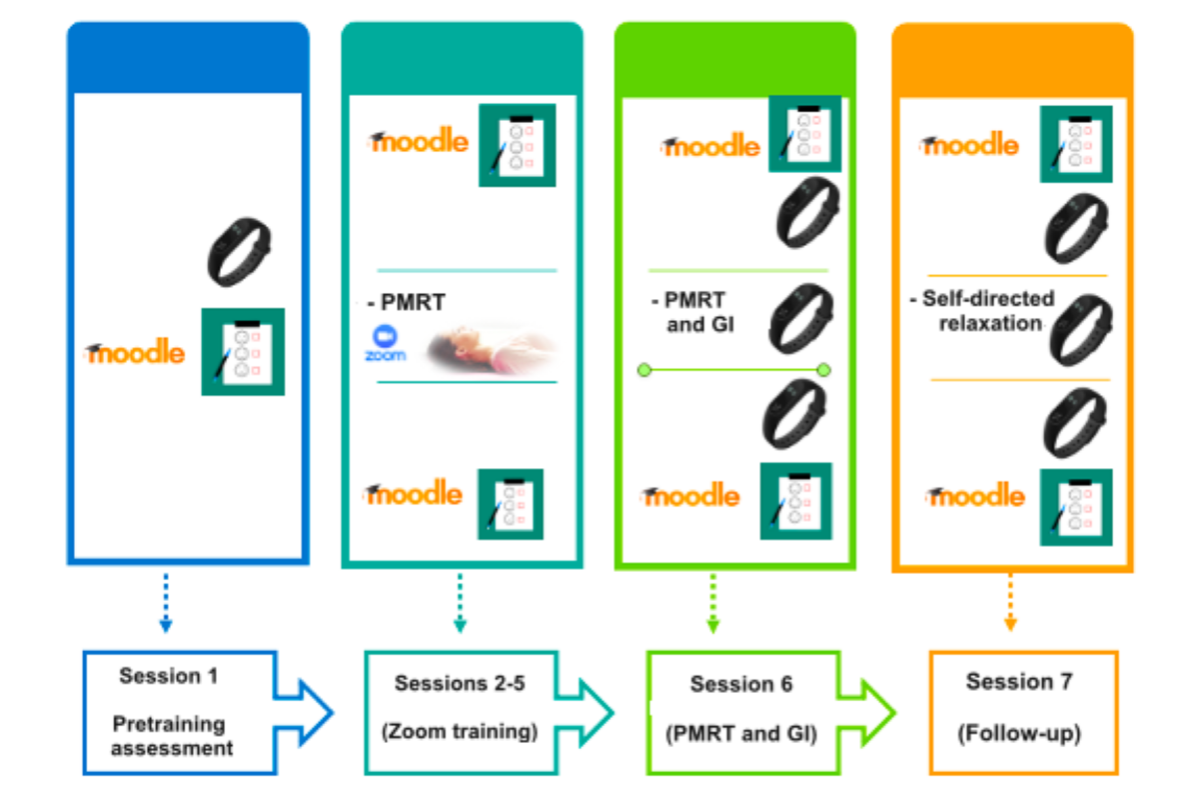
Progressive muscle relaxation technique (PMRT) and guided imagery (GI) exposure.

**Figure 3:**
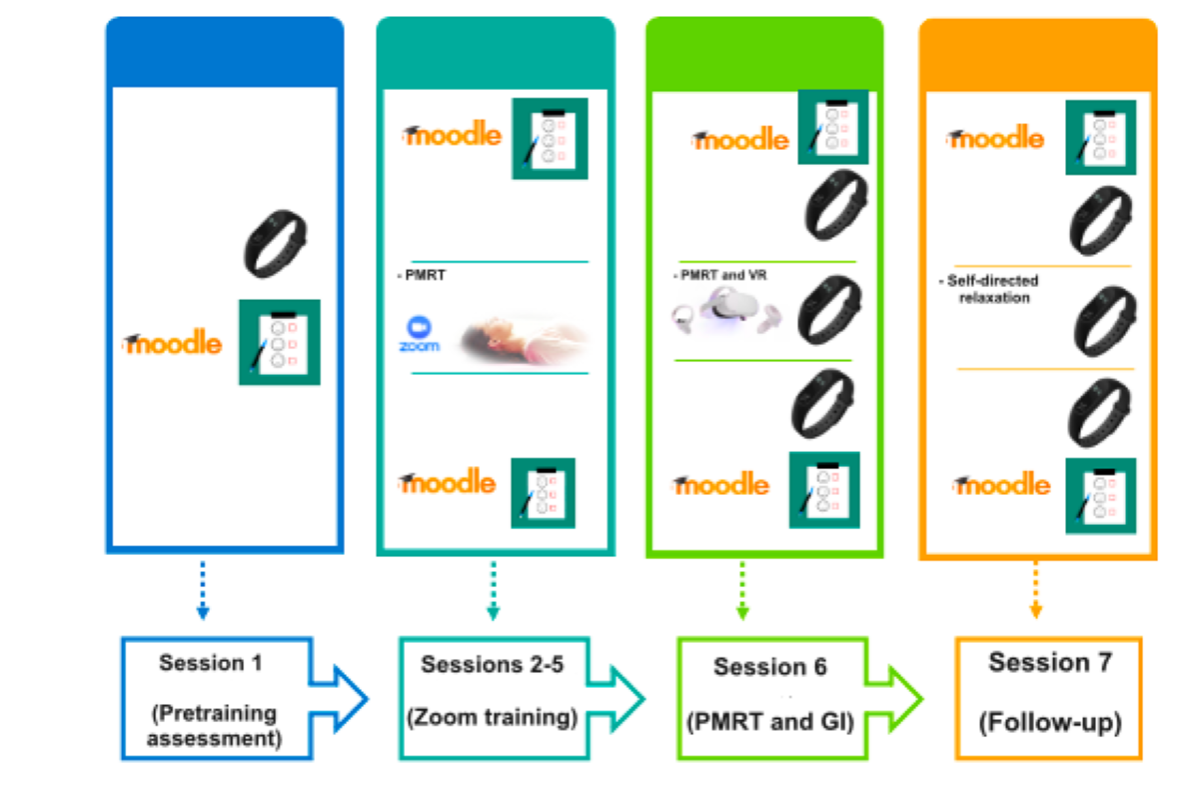
Progressive muscle relaxation technique (PMRT) and personalized virtual reality (VR) exposure.

Specifically, the compared groups’ conditions were characterized as follows: (1) the *PMRT and GI condition* consisted of the deployment of a standard behavioral intervention based on 4 individual active PMRT sessions via Zoom (sessions 2-5; T1-T4), an in vivo PMRT relaxation session with GI conducted by the psychotherapist (session 6; T1) a week after the baseline assessment (session 1; T0), and a follow-up phase (session 7; T2) after 2 weeks consisting of recovering the in-imagination relaxing scenario and a PMRT session (Figure 2); and (2) the *PMRT and VR condition* consisted of 4 active PMRT sessions administered via Zoom (sessions 2-5; T1-T4), a passive PMRT session integrated with the exposure to personalized VR scenarios deployed using the Meta Quest 2 HMD (session 6; T1), and a follow-up phase consisting of the same activities as the PMRT and GI condition (session 7; T2; Figure 3).

The assessment phases were completed via the Moodle open-source learning platform. For this purpose, students could use their institutional accounts to access the platform. The administrator of the experimental procedure then created an ID profile for each participant. To access the Moodle platform, participants did not have to pay.

The assessment phases took place at baseline (T0); before and after each of the 4 active PMRT sessions; before and after the passive PMRT session administered with VR or GI (T1); and a week later, when participants were asked to lead the relaxation session autonomously (T2).

The T0 (baseline) assessment phase lasted approximately 40 minutes; it was the same for all participants and was administered at the VR laboratory at the University of Padova. It involved the administration of the following measures: (1) a demographic schedule [30]; (2) a series of self-report questionnaires investigating depression, anxiety, stress, quality of life, and distress coping strategies (State-Trait Anxiety Inventory–Form Y [STAI-Y]; Depression, Anxiety, and Stress Scale–21 [DASS-21]; Psychological General Well-Being Index [PGWBI]; and Coping Orientation to the Problems Experienced–*Nuova Versione Italiana* [COPE-NVI]); and (3) resting heart rate detection using the Xiaomi Mi Band 2.

Before and after each relaxation session (T1-T4), in approximately 20 minutes, the personal level of tension and relaxation was assessed using a visual analog scale (VAS) from 0 (no tension) to 10 (extreme tension level). The state anxiety level was evaluated based on the STAI-Y1. The 4 relaxation sessions were administered 2 to 3 days apart for all 3 groups. The assessment phase was web-based administered through Moodle, an e-learning platform used for data collection.

The T1 phase (day 7) took approximately 60 minutes and took place at the VR laboratory at the University of Padova. Before and after the relaxation session, states of tension and relaxation and anxiety were assessed using a 0 (no tension) to 10 (extreme tension level) VAS. The state anxiety level was evaluated based on the STAI-Y1. Participants were then exposed to a PMRT session merged with a VR or GI procedure. Before the in-imagination or VR experience, all the participants filled out the Vividness of Visual Imagery Questionnaire and the Test of Visual Imagery Control. After the PMRT session, users filled out a series of self-report questionnaires investigating depression, anxiety, and stress (STAI-Y and DASS-21), and only the VR group filled out the Virtual Reality Symptom Questionnaire (VRSQ) to monitor VR-related side effects (eg, sickness) and the International Test Commission–Sense of Presence Inventory (ITC-SOPI) to assess the sense of presence at the end of the T1 phase. The Xiaomi Mi Band 2 was used during the entire T1 phase to detect resting heart rate activity. The assessment phase was administered through the Moodle e-learning platform.

The T2 phase was deployed for approximately 45 minutes at the VR laboratory at the University of Padova. Before and after the relaxation session, states of tension and relaxation and anxiety were assessed using a VAS from 0 (no tension) to 10 (extreme tension level). The state anxiety level was evaluated based on the STAI-Y1. All users were exposed to a self-GI experience in which those who were part of the VR group were asked to recall the personalized VR scenario experienced during the T1 phase (day 7). The GI group retrieved instead the image that participants had used in association with the PMRT during the T1 phase (day 7). After the session, participants filled out a series of self-report questionnaires investigating depression, anxiety, stress, and quality of life (STAI-Y, DASS-21, and PGWBI) and an ad hoc version based on the ITC-SOPI to assess the sense of presence experienced during the imagery session. This assessment phase was administered based on the Moodle e-learning platform. The Mi Band 2 was used during the entire T1 phase administration (day 7) to detect resting heart rate activity.

### Sample Size Estimation

**Figure 4:**
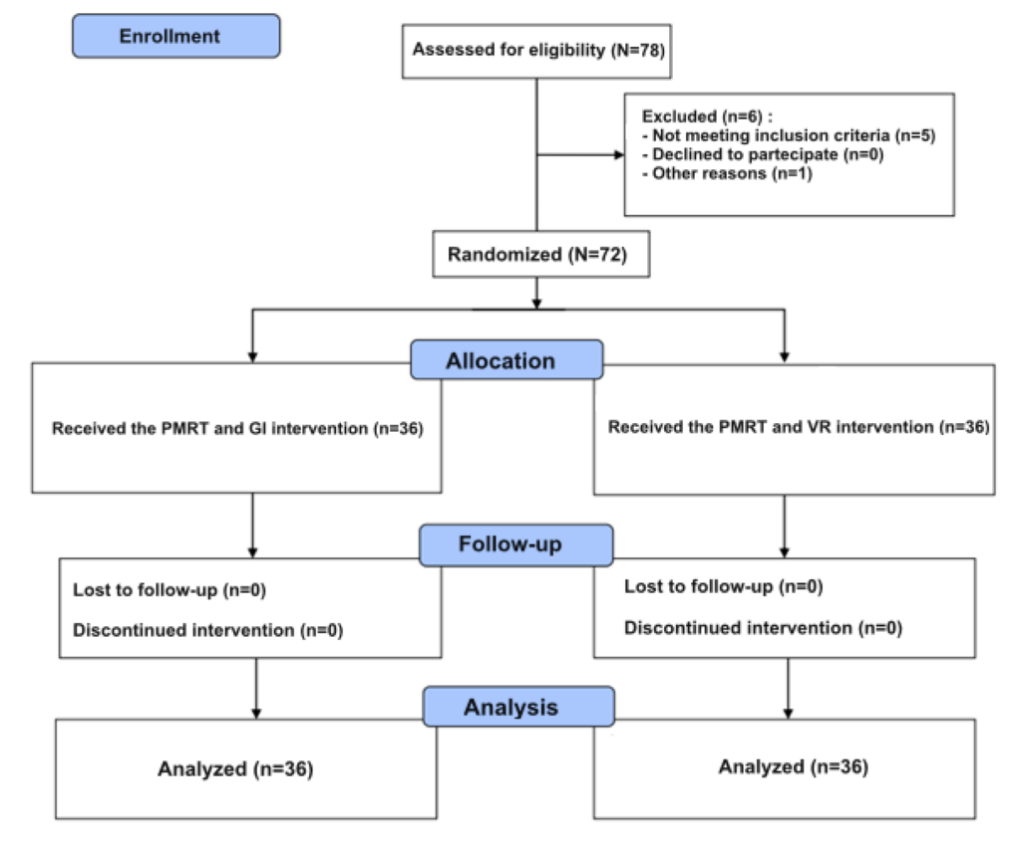
CONSORT flow diagram. Progressive muscle relaxation technique (PMRT) and guided imagery (GI) condition: “As usual” intervention; PMRT and virtual reality (VR) condition: “Experimental” intervention.

To investigate whether the parameters based on our sample size could be acceptable, a formal sample size calculation was conducted using the G*Power (version 3.1) software [31]. As a statistical test, the repeated-measure ANOVA between the factors was considered. The effect size was 0.28, the Cronbach α was .05, and the power (1 − β error probability) was 0.80. We had 2 separate groups and 3 measurements. On the basis of these parameters, it was estimated that at least 35 participants should be recruited for each group. The enrollment process is summarized in Figure 4.

### Statistical Analysis

Quantitative statistical analyses were conducted using the SPSS (version 29.0; IBM Corp) software [32]. Frequencies, means, and SDs were measured to explore the sociodemographic features.

To examine the between- and within-subject differences, the 2 groups were evaluated using a repeated-measure multivariate analysis of covariance (MANCOVA) for mixed designs, multivariate MANCOVA, and *t* tests. To compare the differences between and within groups, pairwise comparisons with Bonferroni CI adjustment were calculated. Multiple linear regression analyses were conducted to investigate whether the sense of presence, engagement, and perception that the scenario was realistic could be predictive of the state anxiety level experienced after the VR exposure.

### Outcome Measures

The measures were administered before and after each relaxation session. The heart rate frequency was recorded based on the Xiaomi Mi Band 2 before the session to obtain baseline data during and after the trials.

A sociodemographic schedule was filled out to obtain information about gender; age; mother tongue; marital status; years of education; occupation; psychological, medical, and neuromuscular problems; use of drugs; and whether participants had relaxation training experience or had used VR devices in the past.

The STAI-Y [33-35] is a self-report questionnaire that allows for the investigation of state and trait anxiety using 40 items on a 4-point Likert scale. Items are grouped into 2 scales focused on how participants generally feel (trait anxiety) or what they experience at particular times (state anxiety). The reliability and validity of the STAI-Y are good in the Italian sample. This study highlighted a moderate to acceptable internal consistency for the state and trait anxiety scales administered (.70<Cronbach α<.84).

The VAS for the tension and relaxation level is a measure of tension and relaxation intensity administered before and after each VR experience. Participants had to express how tense and activated they felt (0=*not at all*; 10=*completely*). Lower scores indicated higher relaxation levels.

The DASS-21 [36-38] is a self-report questionnaire based on 21 items that provides information about anxiety, depression, and stress symptomatology on a 4-point Likert scale from 0 to 3. Internal consistency and convergent, divergent, and criterion-oriented validity are adequate in the original and Italian versions. On the basis of our sample, a moderate to acceptable internal consistency emerged for the total and the 3 subscales (.77<Cronbach α<.87).

The PGWBI [39-41] is a self-report questionnaire consisting of 22 items that provides a general subjective assessment of psychological well-being. It comprises 6 subscales: anxiety, depression, positivity and well-being, self-control, general health, and vitality. The scores for all subscales can be summarized to provide a summary score, which reaches a maximum of 110 points representing the best achievable “well-being.” The tool’s psychometric properties are good for the original version and Italian validation. Considering our sample, an acceptable internal consistency emerged for the total score (Cronbach α=.72).

The COPE-NVI [42] is a 60-item self-report questionnaire on a 5-point Likert scale that investigates how often people use certain coping strategies with stressful or difficult events. Items are grouped into 5 subscales referring to different coping strategies: social support, avoidance strategies, positive attitude, problem-solving, and turning to religion. This tool is psychometrically valid to measure coping styles in the Italian context. Considering our sample, an acceptable internal consistency emerged for the total and the 6 subscales (.72<Cronbach α<.89).

The Vividness of Visual Imagery Questionnaire [43-46] comprises 16 items on a 5-point Likert scale and investigates individual differences regarding the ability to imagine visual contexts vividly. The participant is asked to generate 4 mental images and evaluate their vividness. On the basis of our sample, a good internal consistency was observed (Cronbach α=.89).

The Test of Visual Imagery Control [45-47] is a measure that evaluates individual differences in the ability to control and modify mental images intentionally. For example, participants are asked to visualize a car and then transform the image according to 10 different descriptions. Responses are recorded. On the basis of our sample, a good internal consistency was found (Cronbach α=.86).

The ITC-SOPI [48,49] is a questionnaire consisting of 42 items on a 5-point Likert scale that allows for the investigation of the sense of presence experienced in a VR context. It comprises 4 subscales investigating the sense of physical space, level of engagement experience in the virtual context, ecological validity, and negative effects of exposure. On the basis of our sample, a good internal consistency was found for all subscales (.67<Cronbach α<.78).

The VRSQ [50,51] assesses the general and eye-related physical symptoms of exposure to a VR environment. The score assigned to each item ranges from 0 to 6, with a maximum total score of 84 (48 for general symptoms and 36 for eye symptoms). Higher scores represent worse symptoms, with 0 corresponding to no adverse effects and 84 to serious adverse effects.

### Ethical Considerations

The proposed study protocol was approved by the institutional review board of the Interdepartmental Ethical Committee of Psychology (17 Area) of the University of Padova (Italy; approval 4701; April 29, 2022). The study complied with the relevant ethical regulations of the Declaration of Helsinki (Italian law 196/2003; European Union General Data Protection Regulation 679/2016).

## Results

### Sociodemographic Features and Comparisons

The sociodemographic features of the study sample are outlined in Table 1.

**Table 1:** Demographic features and comparisons considering psychological constructs.

			Virtual reality group (n=36)		Guided imagery group (n=36)		F test (*df*)		Chi-square (*df*)		η_p_^2^
Gender (women), n (%)			28 (78)		27 (75)		N/A^a^		0.8 (2)		N/A
Age (years), mean (SD)			23.83 (6.10)		30.42 (8.36)		14.56 (1, 69)^b^		N/A		0.17
Years of education, mean (SD)			16.81 (1.31)		18.25 (2.60)		8.87 (1, 69)^b,c^		N/A		0.11
**Marital status, n (%)**							N/A		9.3 (2)^b,d^		N/A
	Single	18 (50)		16 (44)							
	Engaged, noncohabiting	17 (47)		10 (28)							
	Married and cohabiting	1 (3)		10 (28)							
Medication (yes), n (%)			8 (22)		5 (14)		N/A		0.5 (2)		N/A
Psychological problems (yes), n (%)			15 (42)		12 (33)		N/A		0.5 (2)		N/A
Medical problems (yes), n (%)			6 (17)		10 (28)		N/A		1.3 (2)		N/A
Experienced relaxation protocol in the past (yes), n (%)			8 (22)		3 (8)		N/A		2.7 (2)		N/A
Experience with VR^e^ in the past (yes), n (%)			10 (28)		8 (22)		N/A		0.3 (2)		N/A
STAI-Y2^f^ (total), mean (SD)			48.06 (3.78)		47.56 (3.56)		0.96 (1, 69)		N/A		0.05
DASS-21^g^ (anxiety), mean (SD)			3.61 (1.87)		3.69 (1.95)		0.03 (1, 69)		N/A		0.001
DASS-21 (depression), mean (SD)			4.46 (4.09)		5.47 (3.70)		1.18 (1, 69)		N/A		0.02
DASS-21 (stress), mean (SD)			8.56 (3.75)		7.69 (3.00)		1.16 (1, 69)		N/A		0.02
COPE-NVI^h^ (social support), mean (SD)			34.92 (6.36)		32.11 (6.47)		3.44 (1, 69)		N/A		0.05
COPE-NVI (avoidance), mean (SD)			22.83 (4.91)		23.00 (4.04)		0.03 (1, 69)		N/A		0.001
COPE-NVI (positive attitude), mean (SD)			30.78 (5.03)		30.72 (5.26)		0.002 (1, 69)		N/A		0.001
COPE-NVI (problem-solving orientation), mean (SD)			31.97 (5.03)		31.69 (5.27)		0.05 (1, 69)		N/A		0.001
COPE-NVI (transcendental orientation), mean (SD)			17.44 (3.49)		17.31 (3.47)		0.03 (1, 69)		N/A		0.001
PGWBI-22^i^ (total), mean (SD)			60.86 (6.97)		63.52 (9.48)		1.85 (1, 69)		N/A		0.03
VVIQ^j^ (total), mean (SD)			58.53 (9.37)		58.39 (11.21)		0.003 (1, 69)		N/A		0.001
TVIC^k^ (total), mean (SD)			41.56 (5.58)		43.44 (5.32)		2.16 (1, 69)		N/A		0.03

^a^N/A: not applicable.

^b^*P*<.001.

^c^*P*<.01.

^d^*P*<.05.

^e^VR: virtual reality.

^f^STAI-Y2: State-Trait Anxiety Inventory–Form Y2.

^g^DASS-21: Depression, Anxiety, and Stress Scale–21.

hCOPE-NVI: Coping Orientation to the Problems Experienced–*Nuova Versione Italiana*.

^i^PGWBI-22: Psychological General Well-Being Index–22.

^j^VVIQ: Vividness of Visual Imagery Questionnaire.

^k^TVIC: Test of Visual Imagery Control.

A multivariate ANOVA was conducted to investigate whether the groups differed in sociodemographic and psychological characteristics before the relaxation training. A difference between the groups was found only for age, years of education, and marital status (Table 1).

To control for the possible impact that baseline differences could have on the research outcomes, the effects of all these variables were controlled for in the subsequent analyses. The psychological problems referred to by participants were related to problematic relationships with parents, low self-esteem, and relational problems. Moreover, for individuals in the VR group, the VRSQ was administered to assess the possible collateral effects of VR exposure–related nausea. On average, individuals showed light susceptibility to general and eye-related motion sickness levels (VRSQ general score: mean 1.39, SD 1.32, range 0-4; VRSQ eye symptom score: mean 1.56, SD 1.32, range 0-5).

### Objective 1: Investigate Whether PMRT Associated With a Personalized Relaxing Scenario in VR Can Be More Effective in Reducing State Anxiety, Tension and Activation, and Heart Rate Frequency

To investigate the differences between the “Virtual Reality” and “Guided Imagery” groups before and after the complementary relaxation experience in session 6 (T1) and session 7 (T2; Figures 2 and 3), we applied a repeated-measure MANCOVA. No significant within-subject main effect emerged (*F*_2,67_=1.01; *P*=.37). A significant between-subject main effect was found (*F*_1,67_=30.56; *P*<.001), and the VR group showed a greater change after the VR exposure than the GI group did in state anxiety (STAI-Y1; *F*_1,67_=10.27; *P*<.001). After the relaxation experience at T1 (t_67_=−7.82; *P*<.001), the VR group displayed lower state anxiety levels than the GI group both before (t_67_=−2.63; *P*=.01; Cohen *d*=0.91) and after (t_67_=−7.23; *P*<.001; Cohen *d*=2.45) the relaxation session during session 7 (T2; Table 2). Pairwise comparisons between and within the groups were performed before and after the relaxation experience at T1 and T2 (see also Multimedia Appendix 1). After the relaxation sessions at T1 and T2, the VR and GI groups experienced a significant decrease in state anxiety (*P*<.001). Pairwise comparisons within groups highlighted a significant reduction in perceived state anxiety at T1 and T2 for both the VR and GI groups (Table 3).

**Table 2:** Descriptive analysis for group differences.

Dependent variable, time, and group type			Values, mean (SD)
**STAI-Y1^a^ (T1)**			
	**Before^b^**		
		Virtual reality (n=36)	48.06 (3.52)
		Guided imagery (n=36)	50.16 (5.16)
	**After^c^**		
		Virtual reality (n=36)	31.17 (4.54)
		Guided imagery (n=36)	40.00 (4.28)
**STAI-Y1 (T2)**			
	**Before**		
		Virtual reality (n=36)	48.89 (3.04)
		Guided imagery (n=36)	51.78 (3.99)
	**After**		
		Virtual reality (n=36)	32.39 (4.33)
		Guided imagery (n=36)	40.75 (4.74)

^a^STAI-Y1: State-Trait Anxiety Inventory–Form Y1.

^b^Refers to the assessment filled out before the relaxation experience at T1 and T2.

^c^Refers to the assessment filled out after the relaxation experience at T1 and T2.

**Table 3:** Within-group pairwise comparisons (Bonferroni CI adjustment).

Dependent variable, group type, and time				Mean difference (before − after)		SE		*t* test (*df*)		Cohen *d*
**STAI-Y1** ^a^ **(T1)**										
	**Virtual reality (n=36)**									
		Before^b^ and after^c^	17.13^d^		1.07		16.01 (67)		4.15	
	**Guided imagery (n=36)**									
		Before and after	9.93^d^		1.07		9.30 (67)		2.14	
**STAI-Y1 (T2)**										
	**Virtual reality (n=36)**									
		Before and after	16.58^d^		1.10		16.40 (67)		4.41	
	**Guided imagery (n=36)**									
		Before and after	10.69^d^		1.10		10.33 (67)		2.52	

^a^STAI-Y1: State-Trait Anxiety Inventory–Form Y1.

^b^Refers to the assessment filled out before the relaxation experience at T1 and T2.

^c^Refers to the assessment filled out after the relaxation experience at T1 and T2.

^d^*P*<.001.

Regarding the outcomes that emerged from the VAS, no significant differences were observed before the relaxation session at T1 (VR group: mean 3.71, SD 0.37; GI group: mean 4.13, SD 0.37; t_67_=−0.76; *P*=.44; η_p_^2^=0.009), but the groups differed after the T1 session, with higher levels of tension experienced by participants in the GI group (VR group: mean 1.05, SD 0.30; GI group: mean 2.06, SD 0.30; t_67_=−2.23; *P*=.03; η_p_^2^=0.069). The same pattern was observed at T2, with no differences between the groups before T2 (VR group: mean 3.52, SD 0.35; GI group: mean 4.17, SD 0.35; t_67_=−1.24; *P*=.22; η_p_^2^=0.022) and higher scores in tension obtained by the individuals in the GI group after the relaxation session (VR group: mean 2.06, SD 1.71; GI group: mean 2.58, SD 1.80; t_67_=−2.06; *P*=.04; η_p_^2^=0.060). Moreover, the levels of tension decreased in each group after the relaxation session at T1 (VR group: t_67_=5.39, *P*<.001, and Cohen *d*=5.05; GI group: t_67_=4.45, *P*<.001, and Cohen *d*=7.20) and T2 (VR group: t_67_=8.55, *P*<.001, Cohen *d*=7.90; GI group: t_67_=6.68, *P*<.001, and Cohen *d*<6.15).

Heart rate was assessed 3 times (before, during, and after the relaxation exposure). The Mauchly test of sphericity was adequate for the heart rate recorded in both sessions 6 and 7 (*P*=.09). Heart rate frequency was found to have a significant main effect between groups (*F*_1,67_=4.87; *P*=.01) but to be not significant within groups (*F*_2,134_=0.51; *P*=.73). An interaction between the “time of assessment” and “group” factors for heart rate (*F*_2,134_=2.22; *P*=.01) was observed. Specifically, individuals in the VR group recorded lower levels of heart rate during (mean 68.26, SD 5.49) and after (mean 69.11, SD 4.98) the VR exposure at T1 than those of individuals in the GI condition (heart rate during: mean 72.86, SD 5.23; heart rate after: mean 72.22, SD 4.73; −2.79<*t*_1, 67_<−3.60; *P*<.01). No significant outcomes were observed at T2.

### Objective 2: Understand Whether VR Promotes a Better Sense of Presence and Engagement in the Scenario Compared With GI After Session 6 (T1) and Whether it Helps Recall the Image and Be Immersed in the Relaxing Scenario in Session 7 (T2)

Individuals in the VR group reported feeling higher engagement after the experience than participants in the GI group (ITC-SOPI–Engagement: *F*_1,67_=2.85; *P*=.03; η_p_^2^=0.15). Moreover, the VR group participants reported experiencing a more realistic environment than that experienced by individuals who used imagination to create the scenario (ITC-SOPI–Ecological Validity: *F*_1,67_=4.38; *P*=.003; η_p_^2^=0.21). No differences between the groups regarding sense of presence were found (ITC-SOPI–Sense of Presence: *F*_1,67_=1.99; *P*=.11; η_p_^2^=0.11; Table 4).

**Table 4:** Multivariate analysis of covariance.

Dependent variable and group type			Values, mean (SD)
**ITC-SOPI^a^–Sense of Presence (T1)**			
	Virtual reality (n=36)	60.06 (8.64)	
	Guided imagery (n=36)	57.00 (8.99)	
**ITC-SOPI–Engagement (T1)**			
	Virtual reality (n=36)	46.61 (4.66)	
	Guided imagery (n=36)	42.42 (5.71)	
**ITC-SOPI–Ecological Validity (T1)**			
	Virtual reality (n=36)	19.92 (2.12)	
	Guided imagery (n=36)	17.67 (2.75)	
**ITC-SOPI–Sense of Presence (T2)**			
	Virtual reality (n=36)	52.81 (12.56)	
	Guided imagery (n=36)	52.69 (11.55)	
**ITC-SOPI–Engagement (T2)**			
	Virtual reality (n=36)	40.40 (6.88)	
	Guided imagery (n=36)	40.36 (6.16)	
**ITC-SOPI–Ecological Validity (T2)**			
	Virtual reality (n=36)	17.44 (3.85)	
	Guided imagery (n=36)	14.17 (4.06)	

^a^ITC-SOPI: International Test Commission–Sense of Presence Inventory.

To investigate whether VR facilitated image recall during session 7 more than the GI technique did, the sense of presence and engagement experiences were assessed after the relaxation experience in session 7 (T2). Individuals previously exposed to the personalized VR scenario referred to perceiving the same scenario recalled in T2 as more realistic than did individuals that, in the previous session (T1), were exposed to an imagined scenario (ITC-SOPI–Ecological Validity: *F*_1,67_=3.21; *P*=.02; η_p_^2^=0.12). No differences emerged between the groups at session 7 (T2) for sense of presence (ITC-SOPI–Sense of Presence: *F*_1,67_=0.76; *P*=.55; η_p_^2^=0.04) and engagement (ITC-SOPI–Engagement: *F*_1,67_=2.30; *P*=.07; η_p_^2^=0.12; Table 4).

### Objective 3: Investigate Group Differences Regarding Trait Anxiety, Depressive Symptoms, Stress, Coping, and Well-Being

To investigate the differences in trait anxiety, depressive symptoms, and stress between the VR and GI groups at baseline (T0), after session 6 (T1), and after session 7 (T2), we applied repeated-measure MANCOVA. The Mauchly test of sphericity was adequate only for the State-Trait Anxiety Inventory–Form Y2 (*P*>.05). The Hyunh-Feldt correction was adopted for the DASS-21 anxiety subscale. The Greenhouse-Geisser correction was adopted for the DASS-21 depression and stress subscales. A significant interaction between the variables “time of assessment” and “group” factors (*F*_2,140_=3.62; *P*=.02) emerged. The “Virtual reality” group had lower trait anxiety levels than the “Guided Imagery” group after the relaxation session during session 7 (T2; t_67_=−2.43; *P*=.02; Tables 5-7). No significant interactions between “time of assessment” and “group” were found for stress (*F*_1.16,81.41_=1.15; *P*>.05), anxiety (*F*_1.89,132.58_=3.11; *P*>.05), and depressive symptoms (*F*_1.16,81.23_=1.09; *P*>.05) assessed using the DASS-21.

**Table 5:** Descriptive analysis of the State-Trait Anxiety Inventory–Form Y2 (STAI-Y2) at T1 and T2.

Dependent variable, time, and group type				Mean (SD)
**STAI-Y2**				
	**T1**			
		Virtual reality (n=36)	42.75 (3.07)	
		Guided imagery (n=36)	44.11 (4.58)	
	**T2**			
		Virtual reality (n=36)	44.25 (4.07)	
		Guided imagery (n=36)	47.36 (4.29)	

**Table 6:** Between-group pairwise comparisons for the State-Trait Anxiety Inventory–Form Y2 (STAI-Y2; Bonferroni CI adjustment).

Group type		Mean difference (before − after)	SE	*t* test (*df*)	Cohen *d*
**STAI-Y2 (T0)**		0.78	0.98	0.80 (67)	0.14
	Virtual reality (n=36)				
	Guided imagery (n=36)				
**STAI-Y2 (T1)**		−0.17	0.99	−2.18 (67)	−0.35
	Virtual reality (n=36)				
	Guided imagery (n=36)				
**STAI-Y2 (T2)**		−*2.76*^a^	1.13	−2.44 (67)	−0.74
	Virtual reality (n=36)				
	Guided imagery (n=36)				

^a^*P*<.05.

**Table 7:** Within-group pairwise comparisons for the State-Trait Anxiety Inventory–Form Y2 (STAI-Y2; Bonferroni CI adjustment).

Group type and time			Mean difference (before − after)		SE		*t* test (*df*)		Cohen *d*
**Virtual reality (n=36)**									
	T0-T1	*4.83* ^a,b^		0.94		5.14 (67)		1.54	
	T0-T2	*3.77* ^a^		0.84		4.49 (67)		0.97	
	T1-T2	−1.06		0.96		−1.10 (67)		−0.42	
**Guided imagery (n=36)**									
	T0-T1	*3.90* ^a^		0.95		4.41 (67)		0.84	
	T0-T2	0.23		0.84		0.27 (67)		0.05	
	T1-T2	−*3.67*		0.93		−3.95 (67)		−0.73	

^a^*P*<.001.

^b^Italicization indicates that the *P* value is statistically significant.

Differences within and between groups in coping strategies and psychological well-being were investigated at baseline (T0) and after session 7 (T2). No significant differences were found between the “time of assessment” and “group” factors for both the PGWBI questionnaire (*F*_1,70_=0.63; *P*>.05) and all the COPE-NVI subscales (COPE-NVI–Social Support: *F*_1,70_=0.95 and *P*>.05; COPE-NVI–Avoidance Strategies: *F*_1,70_=3.01 and *P*>.05; COPE-NVI–Positive Attitude: *F*_1,70_=3.59 and *P*>.05; COPE-NVI–Problem-Solving: *F*_1,70_=0.28 and *P*>.05; COPE-NVI–Turning to Religion: *F*_1,70_=3.71 and *P*>.05).

## Discussion

### Principal Findings

Research on the use of personalized VR scenarios for promoting relaxation is growing, although few conclusions on their effectiveness have been reached. We know even less about the usefulness of integrating standardized and evidence-based relaxation techniques (eg, PMRT) with new technologies that could promote the independent use by patients, providing a more economical solution to treatment costs. As stated previously, the possibility of personalizing audio and visual stimuli in a VR environment is a promising approach for meeting users’ preferences and needs [22], and it has shown encouraging results in terms of both feasibility and potential impact on psychological well-being [18].

The general aim of this pilot study was to evaluate the impact of a novel complementary relaxation training composed of PMRT and the exposure to a personalized relaxing scenario in VR aimed at reducing anxiety and promoting relaxation in a sample of university students.

First, we were interested in investigating the differences in state anxiety between and within groups in the T1 session, where participants were directly exposed to a virtual or imaginative relaxation session. Although a reduction in state anxiety was observed in both the VR and GI groups, our results highlighted that being in a personalized virtual scenario promoted a more significant reduction in self-perceived state anxiety and tension than being in the imaginative condition. Our results are consistent with those of other studies that showed that VR could be a useful tool in reducing stress and promoting relaxation from an assessment based on self-reported measures [52]. Although the target user sample in the study by Hoag et al [53] comprised children and young adults with acute and chronic illnesses, our results align with their results, showing a significant decline in state anxiety from before to after VR exposure. Our data support the role of VR in facilitating a powerful distraction effect on users, reducing their focus on thoughts and external events that would elicit anxiety responses [54,55].

In addition, our data highlighted how engagement with VR scenarios contributes to reducing perceived anxiety after the VR experience. Indeed, it seems that exposure to the VR scenario instead of the imaginative one facilitates the reduction in anxiety levels after the relaxation session. Even if our results need to be further investigated with larger user samples, they are aligned with those of previous studies that underlined the potential benefit of using pleasant, relaxing, and immersive VR scenarios for facilitating relaxation and engagement in individuals from the general population [18,56,57]. A point of strength of this experimental design is the assessment of the impact that the ability to control and vividly picture mental images may have. As we did not find differences between the 2 groups in these abilities before the exposure to the relaxation session in T1, we can support the observed positive effect that exposure to a VR context can have on reducing anxiety.

Considering the VR group, our data showed similar outcomes regarding state anxiety as those investigated at T2. Indeed, when both groups were asked to self-administer the relaxation session, which consisted of recalling the personalized image they had experienced in VR or imagination at T1, those who had been previously exposed to the personalized VR scenario obtained lower state anxiety scores than those in the other group after session 7 at T2. Considering that individuals in the VR group obtained significantly lower scores both before and after the relaxation session in T2, we confirm the hypothesis that being exposed to the self-managed session at T2 further contributed to lowering the anxiety level in the VR group.

Moreover, the VR group participants perceived the recalled scenario at T2 as more realistic than individuals in the GI group did. In addition, participants in the VR group obtained additional benefits in terms of relaxation and state anxiety reduction related to a more realistic sensorial experience that played a substantial role in facilitating the visualization of the scenario and enabled users to focus their attention on the relaxation activities. Consistent with other studies [55-57], this core outcome confirms the role of immersive VR in promoting relaxation through visualization, engagement, and immersion processes. The prominent impact of being exposed to realistic scenarios in VR on the enhanced visualization at T2 is a key contribution of our study, shedding light on the impact that exposure to VR may also have in more ecological everyday settings, such as when people are not wearing the HMD but have the chance to transfer the relaxation skills learned in VR to real-world situations. Our data are consistent with those of studies that show that a graphical representation of a scenario is more effective in the retention and recall processes than an imaginative representation [58,59].

Even if the aim was to compare VR scenarios with a scene on a PC, our outcome is in line with that of the study by Krokos et al [60], which highlighted the prominent impact of VR scenarios on memory recall ability. The hypotheses of this study and that by Krokos et al [60] are anchored on classic studies in cognitive psychology based on the method of loci [61] and the context-dependent memory theory [62]. These theories imply the essential role of learning and mnemonic processes in creating an association between the mnemonic content and a mental frame of scenarios and then recalling contents by mentally visualizing the scenarios in which the learning and memorization processes took place [60]. As that presence, immersion, and engagement in the VR scenarios imply sensorimotor contingencies similar to those in the real world [63] and the way we create and recall mental constructs is influenced by perception and action in the environment [64,65], our data coherently confirm the potential of immersive virtual environments in enhancing learning and recall for the intervention of vestibular and sensorimotor inputs [66].

Regarding our collected data on heart rate activity, our findings showed a more significant decrease in heart rate frequency in the VR group than in the GI group but only in the session in which participants were directly exposed to 1 of the 2 experimental conditions (at T1) and not when individuals had to self-administer the relaxation session (at T2). We derive from the differences found at T1 that were absent in T2 that the experience in VR was stronger and more engaging than in the condition in which individuals had to recall the immersive image without HMD support. Our data supporting previous studies’ outcomes highlighted the impact of VR relaxing scenarios in maintaining lower levels of heart rate frequency than normal [67], but additional research is needed to deeply investigate whether and how naturalistic and relaxing VR scenarios induce relaxation and stress reduction by providing feedback on changes, for example, in heart rate frequency and variability, respiration rate, or skin conductance.

An interesting result is also related to differences over time and between groups in trait anxiety scores. Indeed, our data showed that, in the VR group, the decrease in trait anxiety scores found at T1 was maintained over time (at T2). Nevertheless, the same was not found for the GI group, in which a decrease in anxiety scores was observed at T1, but it significantly increased again at T2, returning to the baseline values (at T0). The 2 groups did not differ at T1, and both showed positive effects of the relaxation sessions. However, the most interesting fact is that, when anxiety was reevaluated after a week and we asked participants to respond considering how they had generally felt during the previous days, people who had used VR claimed to have a lower level of anxiety than that of participants in the other group. These data also need to be further investigated with more comprehensive samples of participants but support the claim that VR plays a crucial role in amplifying the effectiveness of already validated interventions, maintaining their effect over time.

No differences over time were highlighted in coping strategies and psychological well-being, and this could be because our sample was composed of individuals without a clinical diagnosis and high levels of distress at baseline, or it could even be traced back to the fact that these types of psychological constructs require more sessions and time to highlight an effective change.

### Strengths and Limitations

This contribution adds new knowledge on the importance of customizing and personalizing digital interventions according to the users’ perspectives, needs, and preferences [22]. Another point of strength of this experimental design is the assessment of the impact that the ability to control and vividly picture mental images may have. The outcomes were gathered from a well-designed pilot randomized controlled trial involving 2 selected groups of 72 university students whose sociodemographic and psychological characteristics were controlled for to balance their effect on the variables investigated. The experimental procedure adopted supports the reliability and validity of the results and conclusions presented in this paper. However, this study has some limitations that affect the generalizability of the findings. One limitation is that the results cannot be generalized to the entire nonclinical population as our sample consisted of students from a single university. Moreover, the fact that our sample comprised mainly young female participants may be considered as selection bias as the recruited sample may well be more receptive and motivated to participate in the intervention compared with people with other sociodemographic features [68]. In general, to further generalize and validate our results’ effectiveness, the involvement of participants belonging to clinical and nonclinical populations, stratified according to different sociodemographic characteristics, should be considered. The use of VR can be helpful in overcoming several barriers that standard relaxation procedures present as it is less costly; promotes the availability of relaxing content that could be difficult to generate in the imagination; and is able to promote the sense of presence, immersion, and engagement closer to what can be obtained in a real-world situation but in a safer context. This is also a reason for considering its further investigation based on clinical and nonclinical samples in future studies. The preliminary results of our study highlight the potential of VR in reducing the number of psychotherapy sessions and their cost as it allows for partial self-management of the treatment.

Furthermore, in the case of patients with medical problems, the integration of VR could facilitate the administration of the relaxation intervention during specific invasive treatments (such as chemotherapy). If the merged administration of a customized VR relaxation scenario and PMRT is effective in obtaining a better subjective perception of relaxation than the standard procedure, it could allow the users to relax with greater autonomy. For this reason, assessing the efficacy of the PMRT and GI in alternative ways could extend treatment administration, especially in situations in which the standard procedure is more challenging. Future studies should consider structuring the relaxation protocol with more VR sessions to better understand the impact of a virtual scenario on relaxation. Another aspect to be considered in further experimental designs is the introduction of a control group that receives the training session with the physical presence of a therapist. Considering the impact of customization on anxiety and on engaging users in the virtual scenarios, another important aspect that needs to be considered is the opportunity to introduce customized stimuli based on the personal life experience of each participant in the virtual environment. As an example, introducing olfactory stimuli could be another essential aspect to enhance the sense of realism, immersion, and presence in the virtual scenarios [69,70]. A further limitation of this study is the possible exclusion of people who are not familiar with social media. Another limitation regards the limited use of objective data (eg, psychophysiological outcomes) for measuring anxiety and relaxation and the limited range of customization settings offered to users to adapt the VR environment to their preferences. The limitations of this study can be overcome through further investigation.

### Conclusions

The main objective of psychological interventions is to offer people the opportunity to learn strategies to manage their daily lives independently and effectively. The opportunity to deploy VR for promoting self-management of state anxiety also in real-world situations constitutes an interesting line of investigation to be further explored in future studies involving larger nonclinical and clinical populations (eg, patients with chronic pain) to validate and standardize relaxation protocols integrated with new VR tools. This study has shown that personalized VR scenarios can be effective in improving relaxation and decreasing anxiety when integrated with the PMRT as a complementary relaxation method, thereby highlighting the need for further investigation.

## Appendix

Multimedia Appendix 1 Between-group pairwise comparisons (Bonferroni CI adjustment).

Multimedia Appendix 2 CONSORT-EHEALTH (Consolidated Standards of Reporting Trials of Electronic and Mobile Health Applications and Online Telehealth) checklist (V 1.6.1).

## Abbreviations

COPE-NVI: Coping Orientation to the Problems Experienced–Nuova Versione Italiana
DASS-21: Depression, Anxiety, and Stress Scale–21
GI: guided imagery
HMD: head-mounted display
ITC-SOPI: International Test Commission–Sense of Presence Inventory
MANCOVA: multivariate analysis of covariance
PGWBI: Psychological General Well-Being Index
PMRT: progressive muscle relaxation technique
STAI-Y: State-Trait Anxiety Inventory–Form Y
STAI-Y1: State-Trait Anxiety Inventory–Form Y1
VAS: visual analog scale
VR: virtual reality
VRSQ: Virtual Reality Symptom Questionnaire

## Data Availability

The data set generated and analyzed during this study is available from the corresponding author upon reasonable request.

## References

[1] Bayram N, Bilgel N (2008). The prevalence and socio-demographic correlations of depression, anxiety and stress among a group of university students. Soc Psychiatry Psychiatr Epidemiol.

[2] Wiegner L, Hange D, Björkelund C, Ahlborg Jr G (2015). Prevalence of perceived stress and associations to symptoms of exhaustion, depression and anxiety in a working age population seeking primary care--an observational study. BMC Fam Pract.

[3] Son C, Hegde S, Smith A, Wang X, Sasangohar F (2020). Effects of COVID-19 on college students' mental health in the United States: interview survey study. J Med Internet Res.

[4] Komariah M, Ibrahim K, Pahria T, Rahayuwati L, Somantri I (2022). Effect of mindfulness breathing meditation on depression, anxiety, and stress: a randomized controlled trial among university students. Healthcare (Basel).

[5] Ozamiz-Etxebarria N, Dosil Santamaría M, Eiguren Munitis A, Picaza Gorrotxategi M (2020). Corrigendum: reduction of COVID-19 anxiety levels through relaxation techniques: a study carried out in northern Spain on a sample of young university students. Front Psychol.

[6] Baird CL, Sands L (2004). A pilot study of the effectiveness of guided imagery with progressive muscle relaxation to reduce chronic pain and mobility difficulties of osteoarthritis. Pain Manag Nurs.

[7] Nasiri S, Akbari H, Tagharrobi L, Tabatabaee AS (2018). The effect of progressive muscle relaxation and guided imagery on stress, anxiety, and depression of pregnant women referred to health centers. J Educ Health Promot.

[8] Lang PJ (1979). Presidential address, 1978. A bio-informational theory of emotional imagery. Psychophysiology.

[9] Benson H, Beary JF, Carol MP (1974). The relaxation response. Psychiatry.

[10] Syrjala KL, Abrams JR, Gatchel RJ, Turk DC (1996). Hypnosis and imagery in the treatment of pain. Psychological Approaches to Pain Management: A Practitioner's Handbook.

[11] O'Neill LM, Barnier AJ, McConkey K (2006). Treating anxiety with self‐hypnosis and relaxation. Contemp Hypn.

[12] Arena JG, Blanchard EB, Gatchel RJ, Turk DC (1996). Biofeedback and relaxation therapy for chronic pain disorders. Psychological Approaches to Pain Management: A Practitioner's Handbook.

[13] Bakke AC, Purtzer MZ, Newton P (2002). The effect of hypnotic-guided imagery on psychological well-being and immune function in patients with prior breast cancer. J Psychosom Res.

[14] Myers CD, Robinson ME, Guthrie TH, Lamp SP, Lottenberg R (1999). Adjunctive approaches for sickle cell chronic pain. Complement Health Pract Rev.

[15] De Gauquier L, Brengman M, Willems K, Van Kerrebroeck H (2018). Leveraging advertising to a higher dimension: experimental research on the impact of virtual reality on brand personality impressions. Virtual Real.

[16] Lau KW, Lee PY (2018). Shopping in virtual reality: a study on consumers’ shopping experience in a stereoscopic virtual reality. Virtual Reality.

[17] Browning MH, Shin S, Drong G, McAnirlin O, Gagnon RJ, Ranganathan S, Sindelar K, Hoptman D, Bratman GN, Yuan S, Prabhu VG, Heller W (2023). Daily exposure to virtual nature reduces symptoms of anxiety in college students. Sci Rep.

[18] Pardini S, Gabrielli S, Dianti M, Novara C, Zucco GM, Mich O, Forti S (2022). The role of personalization in the user experience, preferences and engagement with virtual reality environments for relaxation. Int J Environ Res Public Health.

[19] Pizzoli SF, Triberti S, Monzani D, Mazzocco K, Kufel E, Porebiak M, Pravettoni G (2019). Comparison of relaxation techniques in virtual reality for breast cancer patients. Proceedings of the 5th Experiment International Conference.

[20] Mazgelytė E, Rekienė V, Dereškevičiūtė E, Petrėnas T, Songailienė J, Utkus A, Chomentauskas G, Karčiauskaitė D (2021). Effects of virtual reality-based relaxation techniques on psychological, physiological, and biochemical stress indicators. Healthcare (Basel).

[21] Ch NA, Ansah AA, Katrahmani A, Burmeister J, Kun AL, Mills C, Shaer O, Lee JD (2023). Virtual nature experiences and mindfulness practices while working from home during COVID-19: effects on stress, focus, and creativity. Int J Hum Comput Stud.

[22] Pizzoli SF, Mazzocco K, Triberti S, Monzani D, Alcañiz Raya ML, Pravettoni G (2019). User-centered virtual reality for promoting relaxation: an innovative approach. Front Psychol.

[23] Holland AC, Kensinger EA (2010). Emotion and autobiographical memory. Phys Life Rev.

[24] Frazier P, Liu Y, Selvey A, Meredith L, Nguyen-Feng VN (2023). Randomized controlled trials assessing efficacy of brief web-based stress management interventions for college students during the COVID pandemic. J Couns Psychol.

[25] Pardini S, Gabrielli S, Olivetto S, Fusina F, Dianti M, Forti S, Lancini C, Novara C (2023). Personalized, naturalistic virtual reality scenarios coupled with web-based progressive muscle relaxation training for the general population: protocol for a proof-of-principle randomized controlled trial. JMIR Res Protoc.

[26] Freesound.

[27] Bernstein DA, Carlson CR, Schmidt JE, Lehrer PM, Woolfolk RL, Sime WE (2007). Progressive relaxation: abbreviated methods. Principles and Practice of Stress Management, 3rd Edition.

[28] Basso JC, McHale A, Ende V, Oberlin DJ, Suzuki WA (2019). Brief, daily meditation enhances attention, memory, mood, and emotional regulation in non-experienced meditators. Behav Brain Res.

[29] Rooks JD, Morrison AB, Goolsarran M, Rogers SL, Jha AP (2017). “We are talking about practice”: the influence of mindfulness vs. relaxation training on athletes’ attention and well-being over high-demand intervals. J Cogn Enhanc.

[30] Pawlow LA, Jones GE (2005). The impact of abbreviated progressive muscle relaxation on salivary cortisol and salivary immunoglobulin A (sIgA). Appl Psychophysiol Biofeedback.

[31] Faul F, Erdfelder E, Buchner A, Lang AG (2009). Statistical power analyses using G*Power 3.1: tests for correlation and regression analyses. Behav Res Methods.

[32] (2022). IBM SPSS Statistics for Windows. IBM Corp.

[33] Spielberger CD, Gorsuch RL (1983). Manual for the State-trait Anxiety Inventory (Form Y).

[34] Spielberger CD (1989). State-Trait Anxiety Inventory A Comprehensive Bibliography. 2nd edition.

[35] Pedrabissi L, Santinello M (1989). State-Trait Anxiety Inventory - Forma Y.

[36] Lovibond SH, Lovibond PF (1995). Manual for the Depression Anxiety Stress Scales. 2nd edition.

[37] Lovibond PF, Lovibond SH (1995). The structure of negative emotional states: comparison of the Depression Anxiety Stress Scales (DASS) with the Beck Depression and Anxiety Inventories. Behav Res Ther.

[38] Bottesi G, Ghisi M, Altoè G, Conforti E, Melli G, Sica C (2015). The Italian version of the Depression Anxiety Stress Scales-21: factor structure and psychometric properties on community and clinical samples. Compr Psychiatry.

[39] Dupuy HJ, Wenger NK, Mattson ME, Furburg CD, Elinson J (1990). The Psychological General Well-being (PGWB) index. Assessment of Quality of Life in Clinical Trials of Cardiovascular Therapies.

[40] Grossi E, Mosconi P, Groth N, Niero M, Apolone G (2002). [Il Questionario Psychological General Well Being] Questionario per la valutazione dello stato generale di benessere psicologico. Versione Italiana.

[41] Grossi E, Groth N, Mosconi P, Cerutti R, Pace F, Compare A, Apolone G (2006). Development and validation of the short version of the Psychological General Well-Being Index (PGWB-S). Health Qual Life Outcomes.

[42] Sica C, Magni C, Ghisi M, Altoè G, Sighinolfi C, Chiri LR, Franceschini S (2008). Coping orientation to problems experienced-Nuova versione Italiana (COPE-NVI): uno strumento per la misura degli stili di coping. Psicoter Cogn e Comportamentale.

[43] Marks DF (1973). Visual imagery differences in the recall of pictures. Br J Psychol.

[44] Marks DF (1989). Construct validity of the Vividness of visual imagery questionnaire. Percept Mot Skills.

[45] Antonietti A, Crespi M (1995). Analisi di tre questionari per la valutazione della vividezza dell'immagine mentale [Analysis of three questionnaires for assessing the vividness of mental image]. Department of Psychology, Catholic University of The Sacred Heart. Technical report.

[46] Iachini T, Maffei L, Masullo M, Senese VP, Rapuano M, Pascale A, Sorrentino F, Ruggiero G (2019). The experience of virtual reality: are individual differences in mental imagery associated with sense of presence?. Cogn Process.

[47] Gordon R (1949). An investigation into some of the factors that favour the formation of stereotyped images. Br J Psychol Gen Sect.

[48] Lessiter J, Freeman J, Davidoff JB, Keogh E (2001). A cross-media presence questionnaire: the ITC-sense of presence inventory. Presence (Camb).

[49] Chirico A, Gaggioli A (2019). When virtual feels real: comparing emotional responses and presence in virtual and natural environments. Cyberpsychol Behav Soc Netw.

[50] Ames SL, Wolffsohn JS, McBrien NA (2005). The development of a symptom questionnaire for assessing virtual reality viewing using a head-mounted display. Optom Vis Sci.

[51] Chirico A, Maiorano P, Indovina P, Milanese C, Giordano GG, Alivernini F, Iodice G, Gallo L, De Pietro G, Lucidi F, Botti G, De Laurentiis M, Giordano A (2020). Virtual reality and music therapy as distraction interventions to alleviate anxiety and improve mood states in breast cancer patients during chemotherapy. J Cell Physiol.

[52] Kim H, Kim DJ, Kim S, Chung WH, Park KA, Kim JD, Kim D, Kim MJ, Kim K, Jeon HJ (2021). Effect of virtual reality on stress reduction and change of physiological parameters including heart rate variability in people with high stress: an open randomized crossover trial. Front Psychiatry.

[53] Hoag JA, Karst J, Bingen K, Palou-Torres A, Yan K (2022). Distracting through procedural pain and distress using virtual reality and guided imagery in pediatric, adolescent, and young adult patients: randomized controlled trial. J Med Internet Res.

[54] McCaul KD, Malott JM (1984). Distraction and coping with pain. Psychol Bull.

[55] Annerstedt M, Jönsson P, Wallergård M, Johansson G, Karlson B, Grahn P, Hansen AM, Währborg P (2013). Inducing physiological stress recovery with sounds of nature in a virtual reality forest--results from a pilot study. Physiol Behav.

[56] Serrano B, Baños RM, Botella C (2016). Virtual reality and stimulation of touch and smell for inducing relaxation: a randomized controlled trial. Comput Human Behav.

[57] Riches S, Azevedo L, Bird L, Pisani S, Valmaggia L (2021). Virtual reality relaxation for the general population: a systematic review. Soc Psychiatry Psychiatr Epidemiol.

[58] Fassbender E, Heiden W (2006). The virtual memory palace. J Comput Inf Syst.

[59] Legge EL, Madan CR, Ng ET, Caplan JB (2012). Building a memory palace in minutes: equivalent memory performance using virtual versus conventional environments with the Method of Loci. Acta Psychol (Amst).

[60] Krokos E, Plaisant C, Varshney A (2018). Virtual memory palaces: immersion aids recall. Virtual Real.

[61] Knauf M (2013). Space to Reason: A Spatial Theory of Human Thought.

[62] Godden DR, Baddeley AD (2011). Context‐dependent memory in two natural environments: on land and underwater. Br J Psychol.

[63] Slater M (2009). Place illusion and plausibility can lead to realistic behaviour in immersive virtual environments. Philos Trans R Soc Lond B Biol Sci.

[64] Barsalou LW (2008). Grounded cognition. Annu Rev Psychol.

[65] Shapiro L (2010). Embodied Cognition.

[66] Hartley T, Lever C, Burgess N, O'Keefe J (2014). Space in the brain: how the hippocampal formation supports spatial cognition. Philos Trans R Soc Lond B Biol Sci.

[67] Varela-Aldás J, Palacios-Navarro G, García-Magariño I, Fuentes EM (2019). Effects of immersive virtual reality on the heart rate of athlete’s warm-up. Proceedings of the 6th International Conference on Augmented Reality, Virtual Reality, and Computer Graphics.

[68] Enzenbach C, Wicklein B, Wirkner K, Loeffler M (2019). Evaluating selection bias in a population-based cohort study with low baseline participation: the LIFE-Adult-Study. BMC Med Res Methodol.

[69] Pizzoli SF, Monzani D, Mazzocco K, Maggioni E, Pravettoni G (2022). The power of odor persuasion: the incorporation of olfactory cues in virtual environments for personalized relaxation. Perspect Psychol Sci.

[70] Abbott RW, Diaz-Artiles A (2022). The impact of digital scents on behavioral health in a restorative virtual reality environment. Acta Astronautica.

