# Supplementary material for: Personalized Virtual Reality Compared With Guided Imagery for Enhancing the Impact of Progressive Muscle Relaxation Training: Pilot Randomized Controlled Trial
Source: JMIR Ment Health. 2024 Jan 30;11:e48649. doi: 10.2196/48649 (PMC10871070; doi:10.2196/48649)
Supplement: Multimedia Appendix 1 [file mental_v11i1e48649_app1.docx]

**Supplementary Materials**

**Table 1 (Supplementary Materials).** Pairwise Comparisons between Groups (Confidence interval adjustment: Bonferroni).

| **Dependent Variables** | **Group type** | **Time** | **Mean Difference (Before - After)** | **Std. Error** | **t-test** | **d-Cohen** |
| --- | --- | --- | --- | --- | --- | --- |
| **STAI-Y1 (T1)** | Virtual Reality  (n=36) | **Before** | -2.02 | 1.05 | -1.92 | -0.48 |
|  | Guided Imagery  (n=36) |  |  |  |  |  |
|  | Virtual Reality  (n=36) | **After** | -9.23*** | 1.18 | -7.82 | -2.00 |
|  | Guided Imagery  (n=36) |  |  |  |  |  |
| **STAI-Y1 (T2)** | Virtual Reality  (n=36) | **Before** | -2.53* | 0.96 | -2.64 | -0.81 |
|  | Guided Imagery  (n=36) |  |  |  |  |  |
|  | Virtual Reality  (n=36) | **After** | -8.77*** | 1.21 | -7.25 | -1.84 |
|  | Guided Imagery  (n=36) |  |  |  |  |  |

**Notes**: * = *P* <.05; ** = *P* <.01; *** = *P* <.001; Before= It refers to the assessment filled out before the relaxation experience at T1 and T2; After= It refers to the assessment filled out after the relaxation experience at T1 and T2.
